# Usability application of multiplex polymerase chain reaction in the diagnosis of microorganisms isolated from urine of patients treated in cancer hospital

**DOI:** 10.2478/raon-2013-0044

**Published:** 2013-07-30

**Authors:** Zefiryn Cybulski, Katarzyna Schmidt, Alicja Grabiec, Zofia Talaga, Piotr Bociąg, Jacek Wojciechowicz, Andrzej Roszak, Witold Kycler

**Affiliations:** 1 Department of Microbiology, Greater Poland Cancer Centre, Poznań, Poland; 2 Genetic Medicine Laboratory CBDNA Research Centre, Poznań, Poland; 3 Department of Radiotherapy and Gynaecologic Oncology, Greater Poland Cancer Centre, Poznań, Poland; 4 Department of Electroradiology, University of Medical Sciences Poznań, Poland; 5 Department of Oncological Surgery II, Greater Poland Cancer Centre, Poznań, Poland

**Keywords:** significant bacteriuria, PCR, microbiological culture, susceptibility tests

## Abstract

**Background:**

The objective of this study was: i) to compare the results of urine culture with polymerase chain reaction (PCR) -based detection of microorganisms using two commercially available kits, ii) to assess antimicrobial susceptibility of urine isolates from cancer patients to chosen antimicrobial drugs and, if necessary, to update the recommendation of empirical therapy.

**Materials and methods.:**

A one-year hospital-based prospective study has been conducted in Greater Poland Cancer Centre and Genetic Medicine Laboratory CBDNA Research Centre in 2011. Urine cultures and urine PCR assay from 72 patients were examined

**Results:**

Urine cultures and urine PCR assay from 72 patients were examined. Urine samples were positive for 128 strains from which 95 (74%) were identical in both tests. The most frequently isolated bacteria in both culture and PCR assay were coliform organisms and *Enterococcus spp*. The Gram negative bacilli were most resistant to cotrimoxazol. 77.2% of these bacilli and 100% of *E. faecalis* and *S. agalactiae* were sensitive to amoxicillin-clavulanic acid. 4.7% of Gram positive cocci were resistant to nitrofurantoin.

**Conclusions:**

The PCR method quickly finds the causative agent of urinary tract infection (UTI) and, therefore, it can help with making the choice of the proper antimicrobial therapy at an early stage. It appears to be a viable alternative to the recommendations made in general treatment guidelines, in cases where diversified sensitivity patterns of microorganisms have been found.

## Intoduction

The most frequently isolated bacteria responsible for urinary tract infection (UTI) are coliform organisms – about 70% of all bacterial strains isolated from urine samples, followed by *Enterococcus spp*. – about 15.0%, coagulase-negative staphylococci -about 10% and *Pseudomonas aeruginosa*- about 5%. Among the coliforms, *E. coli* comprise about 75% of isolates.^1^

UTI may arise as a nosocomial infection and this is the most frequently observed form of this infection.^2^–^4^ The clinical view of UTI can be symptomatic or asymptomatic, all types of the infection may cause serious effects or prolonged stays in hospital. Significant bacteriuria is one of the most important factors in UTI. The immediately introduced empirical UTI therapy may prevent severe complications, for example urosepsis, which is especially dangerous for immunocompromised patients, including cancer patients. Side effects after the treatment of cancer: the soft tissue damage after external radiotherapy or brachytherapy causes serious effects and may have a negative influence for the patient’s immune system.^5^ The most often used antimicrobial drugs for initial, empirical UTI therapy are ciprofloxacin, cotrimoxazole, nitrofurantoin and amoxicillin-clavulanic acid.^6^ The PCR method for the detection of the etiologic infection factor can help when choosing appropriate antimicrobial drugs within a few hours. This rapid method is commonly used in diagnosis of bacteremia.^7^ Therefore, in our study we wanted to investigate two commercially available kits for urine specimen and compare the results with urine culture.

The objectives of the present study were: 1) to compare the results of urine culture with PCR-based detection of urine sample microorganisms and 2) to assess antimicrobial susceptibility of urine isolates to ciprofloxacin, cotrimoxazole, nitrofurantoin and amoxicillin-clavulanic acid and, if necessary, to update the recommendation of empirical therapy of UTI according to results of these analyses.

## Materials and methods

A one-year hospital-based prospective study has been conducted in Greater Poland Cancer Centre and Genetic Medicine Laboratory CBDNA Research Centre in 2011. Urine cultures and urine PCR assay from 72 patients were examined. The study was approved by the ethic commission of University of Medical Sciences Poznan, Poland.

### Urine culture and sensitivity tests

The present analysis was carried out on all urinary specimens with bacteriuria of ≥10^4^ colony forming units (CFU)/mL, including only the first isolate for each patient per two weeks. There was no information on whether the submitted urine samples came from patients with symptomatic upper or lower UTI or asymptomatic bacteriuria. Urine samples were taken as part of the standard patient care and collected with the use of special urine collection system- UriSwab (Copan). The sponge applicators were dipped into urine samples and transported at once to the laboratory in sterile conditions (sterile plastic preservatives). This procedure protected the urine samples from the infection both in the preanalytical and analytical phase as well.

The qualitative and quantitative analysis of urine included urine cultures on the following media for isolation and diagnosis of microorganisms: ChromID CPS chromogenic agar, D-Coccosel medium, Cetrimide agar, Albicans ID2 agar. All ‘under bed’ tests were produced by the firm bioMerieux. Cultures were prepared using quantitative loops and incubated at 35^°^C overnight.

Microorganisms were identified according to standard biochemical tests, which identified most isolated strains to genus level and many to species level. The Vitek identification system (bioMérieux, Marcy l’Etoile, France) was used for confirmation. *In vitro* susceptibility was determined primarily by Vitek AST GP and AST N0 systems.

### Extended-Spectrum Beta-Lactamases (ESBL) detection

For ESBL detection the combined method of disc diffusion with the use of discs with ceftazidime (30 μg), ceftazidime/clavulanic acid (30/10 μg) and cefotaxime (30 μg), cefotaxime clavulanic acid (30/10 μg) was used. The discs were placed on a Mueller Hinton agar plate on which a 0.5 McFarland of test organism was swabbed. An organism was considered to be an ESBL producer if there was ≥ 5 mm increase in zone diameter between the cephalosporins with the clavulanate disc and that of the cephalosporins disc alone.^8^

### DNA isolation

DNA from urine was isolated using NucleoSpin Tissue Macherey – Nagel (MN) firm. Urine samples were collected into the special urine collection system- UriSwab (Copan). The sponge applicators were dipped into urine samples and transported to the laboratory using special plastic preservatives. The sponge applicator absorbed about 2 mL urine. Then 180 μL T1 buffer and 25 μL proteinase K was added to the urine’s sediment and incubated for one hour at 56°C. Then 200 μL of B3 buffer was added and incubated for 10 minutes at 70°C to lyse the samples. 210 μL of 96% ethanol was added to the mixture, which was then loaded onto the column and centrifuged for 1 minute at 11000 g to bind the DNA. To wash the silica membrane, first 500 μL of BW buffer and then 600 μL of B5 buffer was used. This was then centrifuged for 1 minute at 11000 g. The DNA was eluted in 100 μL of TE buffer (Tris-EDTA, pH=8). The DNA concentration was measured using spectrophotometric method by NanoDrop. The concentration of the eluted DNA was between 5–50 ng/μL.

### Multiplex PCR

Multiplex PCR was performed by using two commercially available diagnostic kits: Seeplex UTI ACE Detection and Seeplex Sepsis DNA (Seegene). Both of these kits are intended for diagnosis of urine specimens. The bacteria target genes were identified according to manufacturer’s instructions. The target regions for bacterial genes were not specified by the multiplex PCR producer’s. Using the Seeplex UTI ACE detection kit it is possible to detect the following bacteria: Urophathogenic *E. coli* (UPEC), *Proteus mirabilis, Klebsiella pneumoniae, Staphylococcus saprophyticus, Pseudomonas aeruginosa* and *Enterococcus faecalis.* Seeplex Sepsis DNA kit was used for the detection of *Enterococcus faecium/faecalis*, *Staphylococcus aureus*, *Staphylococcus epidermidis*, *Staphylococcus haemolyticus* and other gram positive cocci from *Staphylococcus spp.* then *Streptococcus agalactiae*, *Streptococcus pneumoniae, Streptococcus pyogenes* and *Streptococcus mitis.*

**FIGURE 1.**
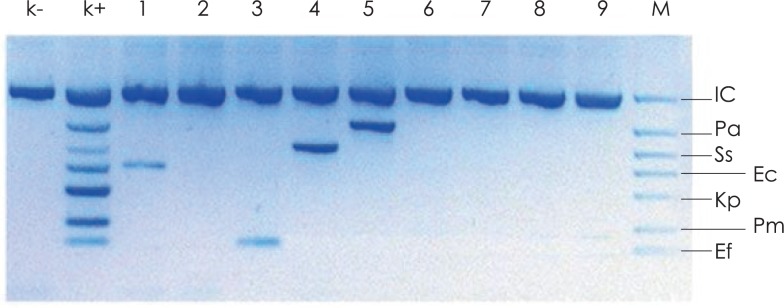
Agarose gel elektrophoresis of PCR amplified products generated from patients DNA urine samples. Lane k- is negative control showing no infection any of the detected pathogens, lane k+ is positive control, lane M is the DNA size marker (UTI DNA ladder supplying by producer). IC = (1000 bp) internal control (DNA plasmid); Pa = (655bp) *P. aeruginosa*; Ss = (526 bp) *S. saprophyticus;* Ec = (401 bp) uropathogenic *E. coli*; Kp = (350 bp) *K. pneumoniae*; Pm = (265 bp) *P. mirabilis*; Ef = (206 bp) *E. faecalis*. Lane 2, 6, 7, 8, 9 shows negative samples, lane 1, 3, 4, 5 shows positive samples: 1 *= E. coli;* 3 *= E. faecalis;* 4 *= S. saprophyticus;* 5 = *P. aeruginosa*

Moreover, this system also detects some gram negative bacteria like: *Enterobacter aerogenes*, *Serratia marcescens, Klebsiella pneumoniae, Enterobacter cloacae, Klebsiella oxytoca, Pseudomonas aeroginosa, Escherichia coli, Proteus mirabilis, Stenotrophomonas maltophilia* and *Acinetobacter baumannii.* The PCR reaction was prepared according to the producer’s instructions. The concentration of the eluted DNA was between 5–50 ng/μL. The PCR solution contained DNA polymerase, dNTPs, MgCl_2_, DNA internal control, primers to internal control and pairs of primers specific to the DNA of the microorganisms. The PCR reaction was amplified in a DNA Engine® thermal cycler (Bio-Rad). 40 thermocycles were performed, each consisting of a 30 s denaturation step at 94°C, a 90 s annealing step at 63°C and a 90 s elongation step at 72°C. In each PCR reaction a positive, negative and internal control were used. The amplified product was visualized under UV light after electrophoresis in a 2% agarose gel containing GelRed (Biotium). Fragment sizes were determined according to marker 100–500 (ABO) (Figure 1).

## Results

### The characteristic of patients group

Urine cultures and urine PCR assay from 72 patients were examined. 60 from them were cancer patients, 31 women and 29 men. The other 12 patients were a heterogeneous group in which cancer was ruled out. The characteristics of the patient group are described in Table 1.

### Comparison of PCR and urine culture results

Urine samples were positive for 128 strains of 20 species of microorganisms from which 95 (74%) were identical in both tests (Table 2).

The multiplex PCR method used here proved to be highly specific since it gave only 3.1% of false positive results in comparison with urine cultures of monomicrobial infections and infections caused with two bacterial strains. It was found that urine samples were infected with one, two or three pathogens (Table 3 and 4). The most frequent were mixed infections of *E. coli* and *Enterococcus spp.* The most frequently isolated bacteria in both culture and PCR assay were coliform organisms from the *Enterobacteriaceae* family: 53 cases (56%) from all bacteria strains, then *Enterococcus spp*.: 26 cases (27%). 17% strains were *Streptococcus spp.* and *Staphyloccus spp. E. coli* constituted 42 strains from culture, including two ESBL positive, which were isolated after two weeks of time interval. Infections caused by only one microorganism were detected in 50 urine samples by PCR and 53 urine samples by culture (Table 3).Two strains were found in 23 urine samples by PCR and 24 urine samples by culture (Table 4).

### The susceptibility of isolates to antimicrobial drugs

Our investigations involved 20 species of microorganisms, including strains of methicillin resistant *S. aureus* (MRSA) and ESBL.

MRSA strain isolated from one urine sample was sensitive to vancomycin, linezolid, quinupristin/dalfopristin, tigecyclin, rifampicin and nitrofurantoin. Besides beta - lactam antibiotics resistance, this strain was resistant to aminoglycosides, tetracyclines, clindamycin and ciprofloxacin, as well.

**FIGURE 2.**
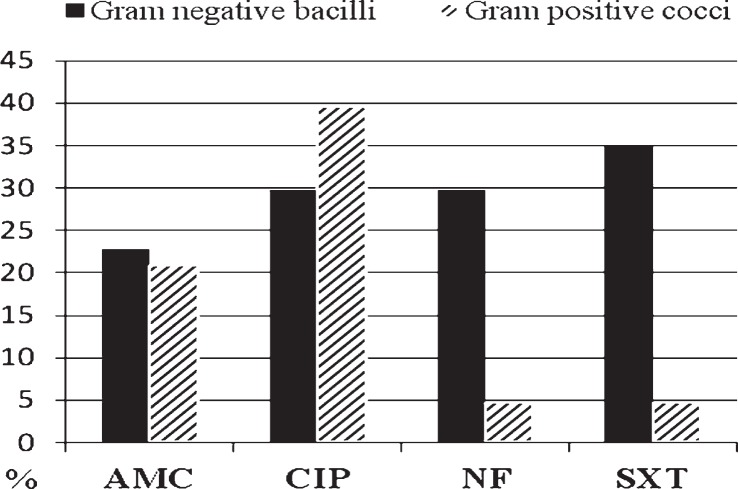
The percentage of strains resistant to antimicrobial drugs. AMC = amoxicillin-clavulanic acid; NF – nitrofurantoin = CIP – ciprofloxacin; SXT = cotrimoxazole

The Gram negative bacilli were most resistant to cotrimoxazol (Figure 2). 29.8% of them were resistant to nitrofurantoin and ciprofloxacin excluding three *P. aeruginosa* strains which were sensitive to chinolons; 77.2% of bacilli and 100% of *E. faecalis* and *S. agalactiae* strains were sensitive to amoxicillin-clavulanic acid. Generally, 4.7% of Gram positive cocci were resistant to nitrofurantoin.

## Discussion

Rapid, sensitive and specific methods for the identification of microorganisms causing urinary tract infections are required at hospitals, in clinical laboratories and for epidemiological purposes. Several urine screening techniques have been described, including Gram stain, quantitative leukocyte counts, direct testing of urine sediment, various biochemical methods and automated systems.^9^ Each of these techniques has many disadvantages limiting significantly their use in a diagnostic laboratory. The urine culture is still the “gold standard” for diagnosis of UTI.^10^,^11^ It is simple and inexpensive. Moreover, many bacteria which are responsible for UTI can easily grow on the medium. However, sensitivity, quality of the medium, risk of interpretation errors by culture and time consuming growing are limitations. The cultures require 24 to 48 hours to provide results after pure cultures are obtained.^12^,^13^ Even with automated systems such a long incubation is necessary and some additional tests may have to be carried out to differentiate species.^12^ The development of molecular techniques has considerably improved the rapidity and accuracy of the microbiological diagnostics. PCR is simple, highly specific, sensitive and amenable to full automation.^14^ It has been successfully used to detect bacterial DNA from different biological fluids. Compared with the classical urine culture, PCR is more rapid and the results are available 5 hours after the specimen collection. However, the use of PCR in diagnostic laboratories is limited by cost, availability of adequate diagnostic kits and availability of appropriate biological materials.^14^ To overcome these shortcomings and to increase the uses of PCR, multiplex PCR has been described.^14^ According to multiplex PCR, which uses several pairs of specific primers to target bacteria sequences, it is possible to detect more than one pathogen in one reaction. It saves considerable time and costs in diagnosis and allows the treatment with a specific antibiotic to be started more quickly.

However, it should be emphasized a need for the critical evaluation of the role of multiplex PCR in the diagnosis of significant bacteriuria. Conventional PCR gives only a presence or absence result of the specific pathogens. On the other hand a small, insignificant number of bacteria may give a positive result in PCR technique. Therefore, a future development of quantitative real time multiplex PCR assays with pathogens specific probes can overcome the limitations of conventional PCR and can be used to quantitative research. In our investigation we used two commercially available kits that are resisted to the dual priming oligonucleotide (DPO) technology (Seegene, Seoul, Korea). This DPO system is structurally and functionally different from the traditional primer available system. It has two separate primer segments with distinct annealing properties incorporated into a single primer, which are joined by poly(I) linker. This blocks the extension of nonspecifically primed templates and achieved consistently high specifically of the assay.^15^,^16^

In the present study multiplex PCR allowed the detection of more pathogens than the cultures, as shown in Tables 2 and 3. According to Lehmann *et al.*, such divergence of the results might be interpreted as false positive PCR assays or false negative microbiological findings.^13^ False positive PCR results can be related to the amplification of free DNA released from unviable or killed bacteria, whereas cultural methods detect only viable and reproductive organisms.^13^ However, two samples with *S. aureus* and one sample with *S. mitis* detected by culture in our investigation showed negative results in PCR. This might be explained by the degradation of DNA.

Karupati *et al.* reported that PCR assays detected low numbers of bacteria in tissues or body fluids that were difficult to culture or that were serologically similar. This might be one of the reasons why multiplex PCR detected more mixed infections than culture.^7^ Knowing that normal human urine microorganisms include numerous opportunistic bacteria and fungi, fastidious and anaerobic microbes, which are potentially pathogenic^17^–^21^, an early detection and identification of etiological factors causing UTI is, therefore, crucial in the clinical setting of the immunocompromised patient.

The diagnosis of UTI is usually based on quantitation of uropathogens in voided urine. Significant bacteriuria is one of the most important. For the criterion of a significant bacteriuria a concentration of ≥10^5^, 10^4^ or 10^3^ CFU/mL may be considered.^22^,^23^ We used ≥10^4^ CFU/mL for purposes of this investigation.

Although microbiological culture takes more time to get results, it is still the best way to generate the sensitivity test which is necessary for the further treatment. The results of antibiotic resistance by PCR are insufficient. Bacterial resistance tests found only mecA gene in MRSA and vanA/ vanB/vanC gene in *Enterococcus spp.* We found one MRSA strain. The infections caused by MRSA are particularly dangerous because of a very high resistance of these bacteria to antibiotics.

PCR method cannot yet replace the traditional microbiological urine culture, but can supplement it and greatly reduce the time needed to obtain results in urgent cases. Rapid and sensitive methods for the identification of pathogens initiated before the empirical therapy may be helpful in choosing the effective treatment, decreasing clinical symptoms and decreasing the proportion of resistant pathogens. Furthermore, the development of DNA based assays may reduce costs by decreasing the length of hospitalization and conserving hospital resources.

Breast and lung cancer are the leading cancer type among women and men, respectively and lung cancer is the most common cause of cancer death worldwide.^24^–^28^ On the other hand, urinary bladder cancer continues to pose a significant global health challenge.^29^ It was most frequently observed cancer between our patients, as well (Table 1).

Choosing the appropriate treatment based on the results of sensitivity tests seemed to be the best means of avoiding the use of unnecessary antibiotics and decreasing the risk of serious complications occurring. There is no publication date suggesting the use PCR method for an early diagnosis of the etiological UTI factor in immunocompromised patients. The described method allows differentiating coliform bacilli from *P. aeruginosa* what is crucial for the proper treatment of patients. *P. aeruginosa* is an opportunistic human pathogen. It is known for its ability to inhabit diverse habitats ranging from soil to immunocompromised individuals.^30^,^31^ We isolated three *P. aeruginosa* strains which were genetically resistant to nitrofurantoin, while 70.2% coliform strains were sensitive to this drug. Because of this the early differentiation between *P. aeruginosa* and coliform is important when nitrofurantoin is introduced into the treatment. Moreover, used PCR methods differentiated *E. faecalis* and *S. agalactiae* that were sensitive to amoxicillin/clavulanic acid from *E. faecium* mostly resistant to this antibacterial drug. Our results draw attention to the emergence of organisms resistant to ciprofloxacin and cotrimoxazole, many authors list drugs as standard treatments for UTI.^6^,^32^ Local UTI treatment guidelines seem to be very important. However, there are substantial differences even between high-standard guidelines on the same well defined clinical entity for UTI management.^33^ The selection of a specific antimicrobial drug for the treatment of a symptomatic UTI episode will be determined by known or suspected susceptibilities of the infecting organism, clinical presentation, patient tolerance, documented efficacy of the agent in the treatment of urinary tract infection, as well as administrative factors such as formulary availability or cost. While the empirical therapy has been initiated, the response to therapy and antimicrobial selection should be re-evaluated after 48 to 72 hours, when culture results are available.^34^,^35^ The results of cultures and sensitivity tests may be used to verify the treatment.

In this study, it was found that most microorganisms remained susceptible to nitrofurantoin (Figure 2). However, this drug was licensed for lower UTIs only, and should be administered for a minimum of 7 days for the empirical therapy.^35^,^36^ Cotrimoxazole was not recommended for the treatment of UTI caused by *Enterococcus sp.* According to our knowledge, there were no available data in the literature concerning rapid diagnostics of bacteriuria in the cancer patients. On the other hand, high risk of serious UTI complications has been well documented in the cancer patients.^37^

## Conclusions

The PCR method quickly identified the causative agent of UTI infection and because of this significantly helped in making the choice of proper antimicrobial therapy at an early stage. It seems it is the better way than recommendation of general treatment guidelines when diversified sensitivity patterns of microorganisms have been found. The multiplex PCR method used here proved to be highly specific since it gave only 3.1% of false positive results in comparison with urine cultures of monomicrobial infections and infections caused with two bacterial strains.

## Figures and Tables

**TABLE 1.** Patient’s characteristics

**Cancer types**	**Number**	**Gender**		**Age (mean age)**	
	
		**Female**	**Male**	**Female**	**Male**
Stomach cancer	2	1	1	71	65
Colorectal cancer	4	3	1	72 (63–80)	64
Uterine cervix cancer	3	3	-	52 (39–66)	-
Prostate cancer	11	-	11	-	67 (56–79)
Brain cancer	2	2	-	44 (20–67)	-
Bladder cancer	15	4	11	73 (64–82)	66 (52–81)
Mamma cancer	4	4	-	51 (42–60)	-
Colon cancer	3	2	1	70 (64–76)	83
Kidney cancer	2	2	-	74 (63–84)	-
Maxilla cancer	1	-	1	-	67
Vagina cancer	1	1	-	76	-
Lymphoma amygdale	2	2	-	64	-
Duodenum cancer	1	1	-	57	-
Lung cancer	1	-	1	-	69
Sigmoid cancer	2	1	1	78	58
Nasopharyngeal cancer	1	1	-	70	-
Pancreatic cancer	1	1	-	78	-
Hepatic cancer	2	1	1	70	63
Thyroid cancer	1	1	-	80	-
Breast cancer	1	1	-	80	-
Non-cancer patient	12	6	6	53 (26–80)	71 (63–80)

**Total**	**72**	**37**	**35**		

**TABLE 2.** Number of microorganisms from urine samples detected in molecular and cultural method. Microorganisms: 1–8 Gram negative bacilli; 9–19 Gram positive cocci; 20 Yeast

**Nr**	**Microorganisms**	**Both tests**	**PCR only**	**Culture only**
1	*E. coli*	41	5	1
2	*K. pneumoniae*	3	1	-
3	*P. mirabilis*	2	1	-
4	*E. aerogenes*	1	1	-
5	*E. cloacae*	3	-	-
6	C. freundii*	-	-	1
7	M. morganii*	-	-	2
8	*P. aeruginosa*	3	4	-
9	*E. faecalis*	20	6	1
10	*E. faecium*	6	-	-
11	E. gallinarum*	-	-	1
12	*S. aureus*	2	-	2
13	*S. haemolyticus*	2	-	-
14	*S. epidermidis*	3	2	-
15	*S. agalactiae*	8	-	-
16	*S. mitis*	1	-	1
17	S. group C*	-	-	1
18	A. viridans*	-	-	1
19	A. hydrophila*	-	-	1
20	C. lusitaniae*	-	-	1

	**Total**	**95**	**20**	**13**

* Pathogens not included in the multiplex panel

**TABLE 3.** Concordance of detectable pathogens in mono- and polymicrobial infections. 1–6 Gram negative bacilli; 7–13 Gram positive cocci

**Nr**	**Pathogen**	**Monomicrobial**	**Infection 2 pathogens**	**Infection 3 pathogens**

		**PCR/culture**	**PCR/culture**	**PCR/culture**
1	*E. coli*	25/23	19/17	2/2
2	*K. pneumoniae*	1/2	1/1	2/0
3	*P. mirabilis*	2/2	0/0	1/0
4	*E. aerogenes*	1/1	0/0	1/0
5	*E. cloacae*	1/1	1/2	1/0
6	*P. aeruginosa*	2/1	2/1	3/1
7	*E. faecalis*	8/8	13/11	5/2
8	*E. faecium*	3/3	3/3	0/0
9	*S. aureus*	0/2	2/2	0/0
10	*S. haemolyticus*	1/2	1/0	0/0
11	*S. epidermidis*	3/3	1/0	1/0
12	*S. agalactiae*	3/5	4/3	1/0
13	*S. mitis*	0/0	1/2	0/0

	**Total**	**50/53**	**48/42**	**17/5**

**TABLE 4.** Number of infected patients detected by culture and PCR

**Methods**	**Patients’ infected 2 pathogens**	**Patients’ infected 3 pathogens**
Culture	24	2
PCR	23	6
